# Epidemiology of COVID‐19 mortality in Nepal: An analysis of the National Health Emergency Operation Center data

**DOI:** 10.1002/puh2.127

**Published:** 2023-10-04

**Authors:** Samir Kumar Adhikari, Kamal Ranabhat, Suraj Bhattarai, Bhuvan Saud, Kiran Paudel, Rabindra Bhandari, Pratik Khanal, Claire Marriott Keene, Vishnu Khanal

**Affiliations:** ^1^ Health Emergency Operation Center Ministry of Health and Population, Ramshah Path Kathmandu Nepal; ^2^ Institute of Medicine Tribhuvan University Kathmandu Nepal; ^3^ London School of Hygiene and Tropical Medicine London UK; ^4^ Global Health Research & Medical Interventions for Development (GLOHMED) Kathmandu Nepal; ^5^ Department of Medical Laboratory Technology Janamaitri Foundation Institute of Health Sciences Lalitpur Nepal; ^6^ Department of Allied Health Sciences University of Connecticut Storrs Connecticut USA; ^7^ Nepal Health Frontiers Kathmandu Nepal; ^8^ Nepal Public Health Association Lalitpur Nepal; ^9^ Bergen Centre for Ethics and Priority Setting in Health (BCEPS), Department of Global Public Health and Primary Care University of Bergen Bergen Norway; ^10^ Health Systems Collaborative NDM Centre for Global Health Research Nuffield Department of Medicine University of Oxford Oxford UK; ^11^ Nepal Development Society (NeDS) Bharatpur Nepal; ^12^ Menzies School of Health Research Alice Springs Northern Territory Australia

**Keywords:** COVID‐19 mortality, epidemiology, global emergencies, Nepal, pandemic preparedness

## Abstract

**Introduction:**

COVID‐19 had caused nearly 12,000 deaths in Nepal by March 2023. In this study, we compare COVID‐19‐associated mortality in the first (September 15 to November 30, 2020) and second (April 15 to June 30, 2021) waves of the pandemic in Nepal and investigate the associated epidemiological factors.

**Methods:**

We disaggregated the COVID‐19‐related deaths between the first and second waves of the pandemic using the national COVID‐19 database and evaluated the association of independent variables with the deaths in the first versus second waves.

**Results:**

Out of 8133 deaths, 25% died in the first wave and 75% in the second. Overall, 33.5% of the deceased were female, and 52% of the deaths were in those 60 years or older. A vast majority (92%) of deaths occurred in hospitals. Geographically, the middle “Hill” region (58.3%) witnessed the most significant number of deaths. About two thirds (64%) had at least one comorbid condition. Multivariable logistic regression showed a difference in the reported deaths by province (state) and geography (ecological region) between the first and second waves. Those in the age groups “19–39 years” and “40–59 years” were more likely to die in the second wave than in the first wave compared to the younger age group.

**Conclusions:**

Overall, deaths were concentrated among older age groups, males, in the Hill regions, in the western provinces, and those with comorbidities. Therefore, the country must focus on these areas to ensure an efficient and effective pandemic response in the future.

## Graphical abstract

The aim of this study was to compare COVID‐19‐associated mortality during the first and second waves. The number of deaths was higher among Hill region, the Bagmati province, the older age group, and those with comorbid conditions. There was significantly higher death in the second wave by geography, administrative region, and age group.

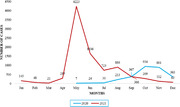

## INTRODUCTION

The COVID‐19 pandemic has had an overwhelmingly greater impact on people's lives than the previous swine flu pandemic in 2009 [1]. No country has remained untouched by this pandemic, with multiple waves causing additional burden [2, 3, 4, 5]. By the end of 2022, over 630 million confirmed cases and nearly 7 million deaths were recorded globally [4]. The severity of COVID‐19 illness varies by factors, such as individual status (age, sex, and race), comorbidity, immune status, and genetic susceptibility [6, 7]. The data has also revealed that the case fatality rate is higher in males than females, possibly because female immune systems generally detect pathogens earlier and have lower numbers of receptors to facilitate viral entry [8]. Individuals with the elevated expression of angiotensin‐converting enzyme (ACE2), transmembrane protease serine 2 (TMPRSS2), and pro‐inflammatory cytokines have been reported to be at a higher risk of the progression of the disease [5]. Moreover, living in overcrowded houses and having lower literacy are also associated with a higher incidence of COVID‐19 [9]. The burden of COVID‐19 is higher in low‐resource settings, with age‐specific mortality rates twofold higher than in high‐income countries [10].

The first COVID‐19 case was reported in a Nepalese man who returned from China in January 2020 [5]. Nepal has since experienced two waves of COVID‐19, with more than 9 million cases, and nearly 12,000 reported deaths up to March 1, 2023 [11]. The country was ill‐prepared for COVID‐19, never having experienced a pandemic of this scale before. Although the effect of the pandemic was experienced at all levels, the impact was much harsher in certain regions of the country due to weaker healthcare systems and competing priorities due to other diseases, lowering the ability to efficiently manage the pandemic. From an evidence perspective, currently available COVID‐19 information is patchy and not representative of the national status. Disaggregated data from different provinces and ecological regions is also not available, which is important as each province and ecological region is unique and has different local contexts that affect the health service availability and utilization. Limited and incomplete data on morbidity and mortality make preparedness and response more challenging. Therefore, this study aimed to investigate the COVID‐19‐associated mortality in the first and second waves of the pandemic in Nepal using a national COVID‐19 mortality database, describe the population who died from COVID‐19, and describe the differences in factors between those dying in the first and second waves, in order to inform future pandemic preparedness.

## METHODS

### Study design

This was a retrospective cross‐sectional study using mortality data from the COVID‐19 digital database, curated by the Health Emergency Operation Center (HEOC), based in the Ministry of Health and Population (MoHP). The dataset includes deaths in Nepal from May 2020 to December 2021.

### Setting

Nepal is a lower middle‐income country with a population of 30.4 million, an annual population growth of 2.3%, a slightly greater female population than male (54% vs. 46%), and a life expectancy of 69 years [11, 12]. Administratively, the country is divided into seven provinces (states) and into three ecological regions (Terai, Hill, and Mountain). The ecological region is based on the altitude and climate of the region [13]. The Southern plain bordering India is the “Terai region.” The middle range with the mountain is the “Hill region,” and the upper Himalayan range, primarily bordering China, is the “Mountain region.” Nepal's health system provides health services through 11 central hospitals, 7 provincial hospitals, 65 district hospitals, primary health centers/health posts at the local government (municipality) level, private hospitals and clinics, and medical colleges and teaching hospitals [12].

In the early phases of the COVID‐19 pandemic, the Government of Nepal (GoN) started 25 hub hospitals within the existing government system, which included COVID‐19‐designated hospitals for the management of symptomatic cases [14]. Later, this was expanded to more than 130 hospitals with the addition of secondary‐level hospitals [15, 16]. Subsequently, the MoHP put forward the concept of “COVID‐19 clinics and level hospitals” and upgraded 125 existing health facilities to COVID‐19 clinics (for fever screening), 23 hospitals to Level‐1 (for isolation of asymptomatic cases), 18 to Level‐2 (for the management of mild/moderate cases), and 3 to Level‐3 hospitals (for the management of severe/critical cases), with some overlaps between the categories [15, 16]. A total of 8000 isolation centers were also established in local communities for asymptomatic COVID‐19 patients and were closely monitored by the local health workers. Locally available schools, community halls, and other buildings were used as isolation centers. In addition to this, 240 holding centers were established for Nepalese migrant returnees at the points of entry (international borders with India and international airport) to closely monitor COVID‐19 symptoms before allowing them to enter Nepal.

The laboratory capacity of the country was insufficient. Even 2 months after the onset of the COVID‐19 pandemic in Nepal, there was only one laboratory in the country, run by the Nepal Public Health Laboratory (NPHL), which was authorized for clinical testing of suspected SARS‐CoV‐2 infection using reverse transcriptase of the polymerase chain reaction (RT‐PCR) technique [17]. At this stage, the samples were collected from different parts of the country and transported to the NPHL (in Kathmandu) for testing. On July 24, 2020, the MoHP approved the “Interim Guidelines for SARS‐CoV‐2 PCR laboratories in the National Public Health Laboratory Network, Nepal,” which outlined the standard operating procedures and requirements for setting up COVID‐19 PCR labs in designated hospitals (hubs, satellites, and level hospitals), including specimen collection, processing, reporting, disposal of specimen, and quality control [18]. The hub laboratories were made responsible for coordinating and supporting their respective satellite laboratories for technical and operational aspects. A protocol was also prepared and implemented, which outlined that people returning to Nepal should be quarantined, either at home or quarantine centers, and tested with a rapid diagnostic test, and then, all suspected cases were to be managed at the hospitals [19]. The GoN rapidly expanded RT‐PCR testing facilities to all 7 provinces, and the number of labs crossed 100 by April 2022 [18, 20]. With the rapid rise of cases and full occupancy of isolation centers, the MoHP advised infected individuals to remain in home isolation for those who were asymptomatic and did not have any complications unless they developed severe symptoms. Isolated patients were under the supervision of local health workers and were able to seek medical help from them. This strategy was adopted to assist already overstretched health facilities and also to reduce possible transmission from one patient to another patient and visitors.

### Dataset

We conducted a retrospective quantitative study using Nepal's National COVID‐19 digital database available from the HEOC [21]. The HEOC works as the secretariat of the MoHP during health emergencies and disasters and is also responsible for data collection and storage. The dataset includes the records of all reported deaths from COVID‐19 between May 2020 and December 2021 in seven provinces of Nepal. It also includes limited demographic variables, comorbidities, and place of death at the individual level. The HEOC developed the database in two phases, initially as a Microsoft Excel–based reporting system, followed by a software‐based reporting system [21]. Such evolution of the HEOC dataset has helped the GoN to eliminate the duplication of reporting systems by various government institutions. The HEOC was able to perform as the “one door” reporting system to avoid such duplication.

### Population

This study included all those who died in Nepal during the first wave (September 15 to November 30, 2020) and during the second wave (April 15 to June 30, 2021).

### Variables

We used the definition of the first and second waves used by the GoN [22] for the purpose of our study. We disaggregated the time frame of COVID‐19‐related deaths into the “first wave” (September 15 to November 30, 2020) and the “second wave” (April 15 to June 30, 2021). The main outcome for the regression was “deaths during the first and second wave” to examine whether there was a significant difference in COVID‐19‐related deaths in the first versus the second waves after controlling for confounding factors available in the database.

A number of independent variables were available in the National COVID‐19 digital database [21]. The age of the deceased was recorded in years, which was later categorized into 0–18, 19–39, 40–59, 60–79, and ≥80 years. Sex was recorded as male or female. Nepal has seven provinces in its current federal structure and was recorded accordingly: Koshi, Madhesh, Bagmati, Gandaki, Lumbini, Karnali, and Sudurpaschim. Each province has its own provincial government with its public health network. Local hospitals and centers work under the direction of the provincial government. It has to be noted that until the COVID‐19 outbreak, some provinces did not have a separate ministry portfolio for health. The ecological region was documented as Mountain, Hill, and Terai. A number of comorbidities were documented: hypertension, type 2 diabetes mellitus, chronic obstructive pulmonary disease (COPD), chronic kidney disease, cardiovascular disease, liver disease, thyroid disorder, cancer, anemia, septic shock, urinary tract infection, stroke, and paralysis, meningitis, hyperkalemia, and thrombosis (Table 2). We categorized comorbidity into a binary variable: “present” if at least one condition was reported, and “absent” if none (Table 3). The place of death was recorded as home, hospital, isolation center, and in transit (to hospital or isolation center).

### Statistical analysis

The data was first extracted from Microsoft Excel and checked for completeness. Subsequently, it was imported into Statistical Package for Social Sciences (SPSS IBM, Ver. 21) for analysis. Individuals with incomplete information were excluded from analysis. The frequency and proportion of the outcome and independent variables were presented. The deaths were classified into “deaths during the first wave (September 15 to November 30, 2020) = 0” and “deaths during the second wave (April 15 to June 30, 2021) = 1” to investigate if the independent variables were associated with the differences in the mortalities. A Chi‐square test was used to assess the association between the outcome variable and independent variables. Multivariable logistic regression was used to ascertain the factors associated with COVID‐19‐associated deaths in the second wave as compared to the first wave. Stepwise backward logistic regression was used to account for the possible interactions among the factors, such as ecological regions, sex, and place of death [23]. Adjusted odds ratios (adjusted OR), together with their corresponding 95% confidence intervals (CI), were reported. A *p*‐value of <0.05 was set as statistically significant.

### Ethical consideration

Ethics approval was obtained from the Nepal Health Research Council Nepal (Reference number 597/2021 P). Administrative approval was also obtained from the MoHP, GoN, to access de‐identified data.

## RESULTS

### Deaths during the first (September 15 to November 30, 2020) and second (April 15 to June 30, 2021) waves

**FIGURE 1:**
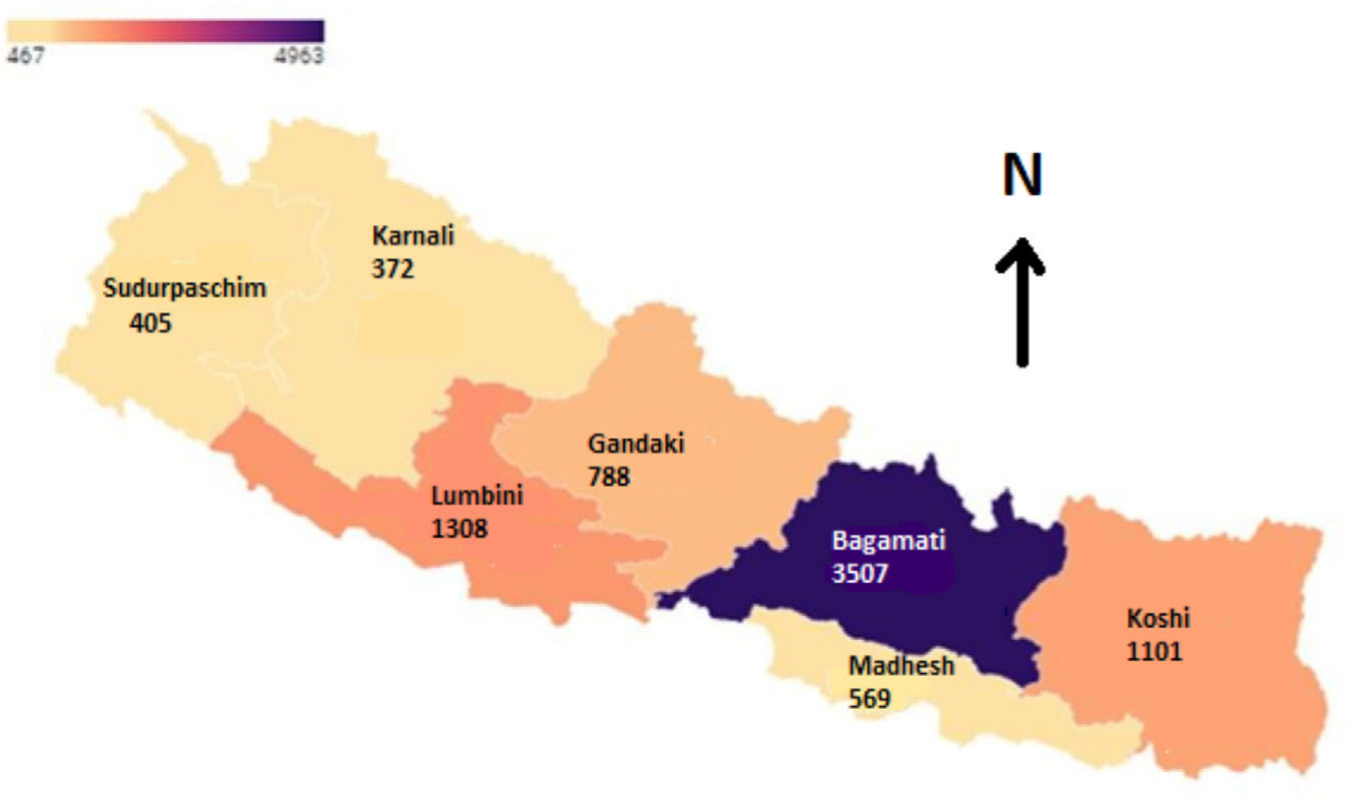
Distribution of COVID‐19 deaths during the first two waves (from May 2020 to December 2021) by province, Nepal.

Table 1 presents the distribution of COVID‐19 deaths during the first and second waves. Out of 8133 deaths, one fourth (25%) died in the first wave, whereas three quarters (75%) died in the second wave. Bagmati Province reported the highest (43%) COVID‐19 deaths overall, followed by Lumbini (17%) and Koshi provinces (13%), as shown in Table 1 and Figure 1. Karnali and Sudurpaschim provinces reported the lowest death during the study period (5% each). Among the ecological regions, the highest deaths were reported in the Hill (61% and 58%), followed by the Terai (37% and 39%) and the Mountain (2% and 4%) regions during both the first and the second waves, respectively.

**TABLE 1:** COVID‐19 deaths by participants characteristics during the first and second waves^a^, Nepal (*n* = 8133).

Characteristics	Total deaths, *n* (%)	First wave, *n* (%)	Second wave, *n* (%)	*p*‐Value
Total	8133	2025 (24.9)	6108 (75.1)	
Province				<0.001
Koshi	1101 (13.5)	377 (18.6)	724 (11.8)	
Bagmati	3507 (43.2)	1017 (50.3)	2490 (40.8)	
Lumbini	1380 (17.0)	255 (12.6)	1125 (18.4)	
Gandaki	788 (9.7)	196 (9.8)	592 (9.7)	
Madhesh	569 (7.0)	113 (5.6)	456 (7.5)	
Karnali	372 (4.6)	19 (0.9)	353 (5.9)	
Sudurpaschim	405 (5.0)	45 (2.2)	360 (5.9)	
**Ecological region**				0.001
Plain region	3113 (38.3)	750 (37.1)	2363 (38.7)	
Mountain	270 (3.3)	45 (2.2)	225 (3.7)	
Hill	4739 (58.4)	1227 (60.7)	3512 (57.6)	
**Age group (years)**				<0.001
0–18	61 (0.7)	25 (1.2)	36 (0.6)	
19–39	950 (11.7)	177 (8.7)	773 (12.7)	
40–59	2860 (35.2)	542 (26.8)	2318 (38.0)	
60–79	3268 (40.2)	955 (47.2)	2313 (37.8)	
≥80	994 (12.2)	326 (16.1)	668 (10.9)	
**Sex**				0.061
Female	2721 (33.5)	643 (31.8)	2078 (34.0)	
Male	5412 (66.5)	1382 (68.2)	4030 (66.0)	
**Place of death**				<0.001
Hospital	7525 (92.5)	1940 (95.8)	5585 (91.4)	
Home Isolation	535 (6.6)	67 (3.3)	468 (7.7)	
Isolation center	34 (0.4)	12 (0.6)	22 (0.4)	
In transit to hospital or isolation center	39 (0.5)	6 (0.3)	33 (0.5)	
**Comorbidity** ^b^				<0.001
Present	1942 (23.9)	832 (91.8)	1110 (52)	
Absent	1098 (13.5)	74 (8.2)	1024 (48)	
Missing data		5093 (62.6)		

*Note*: *p*‐Value: chi‐square *p*‐value.

^a^ First wave: September 15 to November 30, 2020; second wave: April 15 to June 30, 2021.

^b^ Missing values present.

More than half of the deaths (52%) were reported among the elderly population (60+ years). There were fewer deaths in children and adolescents (under 18 years), with less than 1% of total deaths. The majority of deaths (92%) occurred in hospitals, whereas 7% occurred in home isolation. Of 3040 cases that had comorbidity data available during the first and second waves, 64% had at least 1 comorbid condition.

**FIGURE 2:**
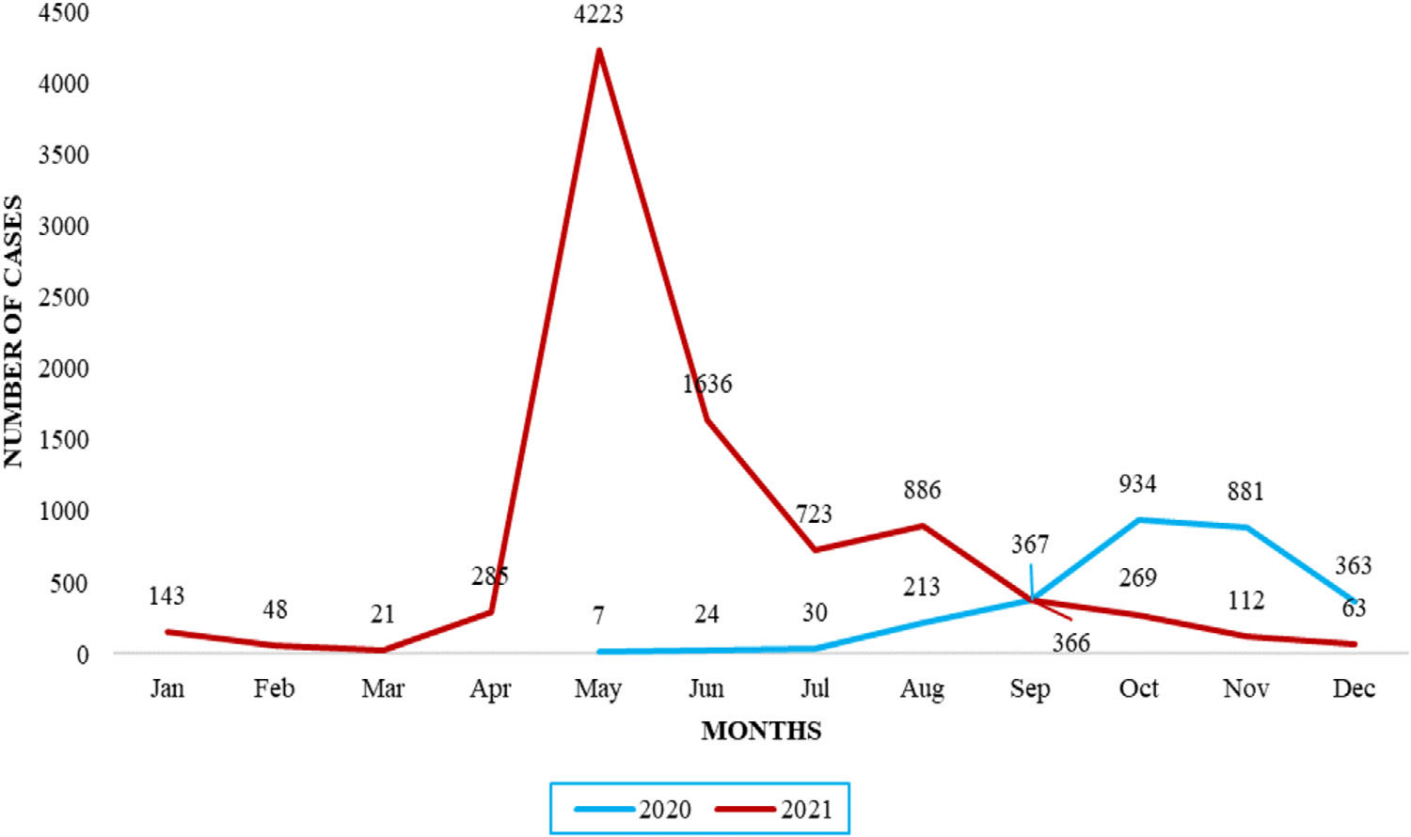
Distribution of COVID‐19 deaths by months in Nepal.

Figure 2 shows the distribution of the number of deaths in 2020 and 2021 by month. A total of 8133 people died of COVID‐19 in Nepal during the first and second waves (Table 1). The first death was reported in Nepal on May 17, 2020, and the highest recorded death rate was in October 2020 of the first wave. In the second wave, May (2021) recorded the highest number of deaths, with more than 4000 deaths in the month alone (Figure 2). The second wave subsided after July 2021, with a slight upward trend again in August and October 2021.

### Comorbidities among the deceased

Table 2 shows the prevalence of comorbidities among the deceased. Hypertension was the leading comorbidity (41%), followed by type 2 diabetes mellitus (28%), respiratory diseases, including COPD (25%), chronic kidney disease (20%), and cardiovascular disease (8%). The proportion of the deceased with comorbidities was greater in the cohort who died in the first wave (92%) than in the second wave (52%).

**TABLE 2:** Frequency of comorbidities among people who died of COVID‐19 from May 2020 to December 2021, Nepal.^a^

Comorbidity^a^	Frequency (*n* = 3439)	Percentage
Hypertension	1407	40.9
Type 2 diabetes mellitus	977	28.4
Chronic kidney disease	688	20.0
Respiratory disease (including COPD)	853	24.8
Cardiovascular disease	271	7.98
Liver disease	180	5.2
Thyroid disorder	96	2.8
Cancer	60	1.7
Anemia	44	1.38
Septic shock	33	1.0
Urinary tract infection (UTI)	32	0.9
Stroke and paralysis	22	0.6
Meningitis	8	0.2
Hyperkalemia	6	0.2
Thrombosis	5	0.1

Abbreviation: COPD, chronic obstructive pulmonary disease.

^a^ Multiple response. Data from May 2020 to December 2021.

### Factors associated with the higher COVID deaths during the second wave (April 15 to June 30, 2021)

The factors associated with higher COVID‐19 deaths during the second wave are presented in Table 3. There was significant geographic variation. The Western states, namely, Lumbini (adjusted OR 1.88; 95% CI: 1.35, 2.63), Karnali (adjusted OR: 5.05; 95% CI: 2.25, 11.34), and Sudurpaschim (adjusted OR: 2.58; 95% CI: 1.33, 5.03), reported more deaths compared to the Eastern state (Koshi). The Hill region of the country (adjusted OR: 1.10; 95% CI: 1.06, 1.34) witnessed more deaths compared to the Terai region in the second wave compared to the first wave. Higher odds of dying in the second wave were reported among 19–39 years (adjusted OR: 3.39; 95% CI: 1.37, 8.39) and 40–59 years (adjusted OR: 3.76; 95% CI: 1.56, 9.05) compared to the age group 0–18 years but were not significant in the over 60‐year age groups. The odds of such difference in mortality in two waves were lower among those who did report having at least one underlying noncommunicable disease (NCD) (adjusted OR: 0.10; 95% CI: 0.08, 0.14).

**TABLE 3:** Factors associated with higher COVID‐19 deaths during the second wave^a^ in Nepal.

Characteristics	Odds ratio (95% confidence interval)	Adjusted odds ratio (95% confidence interval)
**Province**	*p* = 0.001	*p* = 0.001
Koshi	1.00	1.00
Madhesh	2.10 (1.65, 2.67)	1.19 (0.80, 1.78)
Bagmati	1.27 (1.10, 1.47)	1.01 (0.72, 1.42)
Gandaki	1.57 (1.28, 1.93)	0.83 (0.54, 1.25)
Lumbini	2.30 (1.91, 2.76)	1.88 (1.35, 2.63)
Karnali	9.67 (5.99, 15.60)	5.05 (2.25, 11.34)
Sudurpaschim	4.17 (2.98, 5.81)	2.58 (1.33, 5.03)
**Ecological region**	*p* = 0.001	*p* = 0.045
Terai	1.00	1.00
Mountain	1.59 (1.14, 2.12)	1.16 (0.62, 2.07)
Hill	0.91 (0.82, 1.01)	1.10 (1.06, 1.34)
**Age group (years)**	*p* < 0.001	*p* < 0.001
0–18	1.00	1.00
19–39	3.03 (1.77,5.12)	3.39 (1.37, 8.39)
40–59	2.97 (1.76, 4.99)	3.76 (1.56, 9.05)
60–79	1.68 (1.00, 2.82)	2.18 (0.91, 5.22)
≥80	1.42 (0.84, 2.41)	1.65 (0.67, 4.02)
**Comorbidity**	*p* < 0.001	*p* < 0.001
Absent	1.00	1.00
Present	0.11 (0.07, 0.12)	0.10 (0.08, 0.14)

*Note*: Variables controlled in multiple regression model: ecological region, sex, and place of deaths. *p*: *p*‐value.

^a^ First wave: September 15 to November 30, 2020; second wave: April 15 to June 30, 2021.

## DISCUSSION

This study used the national COVID‐19 death registry maintained by the HEOC situated at the MoHP, Nepal. The study found higher COVID‐19 deaths in the second wave (April 15 to June 30, 2021) compared to the first wave (September 15 to November 30, 2020). A number of factors, such as sociodemographic variables and comorbid status, appeared to be associated with dying in the second wave over the first wave.

The number of deaths in the second wave (2021) was three times higher than in the first wave (2020). In the South Asian region, the first wave was comparatively less severe than the subsequent waves [24]. The second wave in Nepal was predominantly caused by the COVID‐19 Delta variant (B.1.617.2), which was more virulent than the variants of SARS‐CoV‐2 that caused that first wave [25, 26]. Additionally, the population may have been more complacent during the second wave. A previous study [25] asserted that people had started returning to their normal routine by April 2021 with a false sense of the “worst was over” belief as the nationwide vaccination rolled out as early as January 2021 [27]. However, in reality, there was a vaccine shortage in the country, and the supply chain was slow [27, 28]. Furthermore, around the onset of the second wave, a major surge in cases and a strict lockdown in India created an influx of migrant Nepalese workers returning to Nepal [29]. Within Nepal, mobility also increased as people started returning to their hometowns from the major cities. Finally, the higher numbers of COVID‐19 deaths in the second wave could also be attributed to improved reporting systems as the pandemic progressed [28].

This analysis highlighted that there was a disproportionate contribution to mortality from different age groups, gender, comorbidity status, and geographic locations. A higher proportion of deaths occurred among the age group 60–79‐year age group compared to their younger counterparts, and this was greater in the first wave than in the second (Table 1). The finding is consistent with the previous findings from Nepal [10], Bangladesh, and India [2]. The higher number of deaths among the older age group may be linked to weaker immune systems among older people [2]. Furthermore, the older cohort of 60–79 years has a higher burden of NCDs compared to their younger counterparts in Nepal. Global data showed that the highest death rate was noted in those with cardiovascular diseases, followed by diabetes, chronic respiratory diseases, hypertension, and cancer [30, 31, 32]. Our study showed that 4 in 10 deceased had hypertension and almost 1 in 3 had type 2 diabetes, compared to the national prevalence of hypertension of 32% (95% CI: 23%–40%) [33] and type 2 diabetes of 8.5% (95% CI: 6.9%–10.4%) [34] among the adult population. The association of NCDs, such as hypertension and type 2 diabetes, and higher COVID‐19 deaths has been well documented [2]; thus, the higher prevalence of the two diseases among the deceased is not surprising. This information is important for future public health programs, given that Nepal is facing the triple burden of emerging infectious diseases, re‐emerging infectious diseases, and NCDs [35]. A tailored approach for those most affected is essential while designing future public health programs.

We noted that there was geographical variation in the number of COVID‐19 deaths overall and between waves. Hill regions had recorded more deaths in both waves than the Terai and Mountains. This may be explained by the fact that Hill regions have cold weather that may prolong the environmental survival of SARS‐CoV‐2, have houses with poor ventilation contributing to increased exposure to COVID‐19 cases, and relatively cooler temperatures prolonging transmission. Such geographical variations are not uncommon and have been reported in neighboring countries such as India and Pakistan [2].

The descriptive finding showed that the highest numbers of deaths were recorded in Bagmati province, the location of the capital city Kathmandu. The dense population in Kathmandu Valley compared to other regions of the country, and exposure to international travel may explain the higher proportion of deaths in the province. A more concentrated population has been linked with higher COVID‐19 deaths in India, Pakistan, and Bangladesh [2]. Provinces, such as Lumbini, Karnali, and Sudurpaschim, which have higher migration and population mobility with India, reported higher numbers of deaths and a greater proportion of deaths in the second wave compared to the first. This indicates that any outbreak in the nearby Indian regions should be taken seriously by the Nepalese government and rapidly prepared for response.

### Strengths and limitations of the study

To our knowledge, this is the first study that has used nationally representative data to explore COVID‐19 deaths during the first and second waves. This study also highlighted the vulnerable areas by age, health status, and geographic location for future pandemic preparedness and response. Notwithstanding the strengths, certain limitations must be acknowledged. We have presented the association of the key independent variables with the deaths during the first and second waves, not between deaths and recovery. We do not have data on the total number of infections and recovery compared with deaths. Therefore, reporting on the incidence rate and identifying factors associated with deaths and/or survival was not possible. We stress that our data needs to be interpreted carefully. Data quality of deaths during emergencies has been criticized in the South Asian region, and the current dataset is likely to have similar limitations. The dataset had significant missing cases on comorbidities among the deceased, which is another limitation of the current study. Likewise, the database only records limited socioeconomic variables, which has restricted our ability to analyze them further. Nevertheless, as the data management skills and expertise evolve, Nepal must expand and improve its death registration and medical certification system.

## CONCLUSION

The pandemic has affected all aspects of the health system. There were more deaths in both waves among the older cohort, males, hill areas, the highly populated Bagmati province, and those with comorbidities. This study also reported higher numbers of deaths in the second wave of the pandemic, with an association with age and geographical location. This provides evidence and motivation to the policymakers that pandemic preparedness and emergency readiness have to be in place for another COVID‐19 wave or similar high‐impact respiratory epidemic. Such readiness will not only reduce the impact of inevitable pandemic in the future but also help manage periodic influenza epidemics within the country.

## AUTHOR CONTRIBUTIONS

Samir Kumar Adhikari and Kamal Ranabhat jointly conceptualized the study and led the study. Rabindra Bhandari and Kiran Paudel analyzed the data with input from Vishnu Khanal. Samir Kumar Adhikari, Kamal Ranabhat, Suraj Bhattarai, and Vishnu Khanal contributed to writing the manuscript. Bhuvan Saud, Pratik Khanal, and Claire Marriott Keene critically reviewed the manuscript and provided substantial input. All authors agreed on the final version of the manuscript.

## CONFLICT OF INTEREST STATEMENT

The authors declare that there are no conflicts of interest that could be perceived as prejudicing the impartiality of the research reported.

## ETHICS STATEMENT AND CONSENT TO PARTICIPATE

Ethical approval for the research was obtained from the Ethical Review Board of the Nepal Health Research Council (NHRC), Kathmandu Nepal (Reference number 597/2021 P). An administrative approval was also obtained from the Ministry of Health and Population, Government of Nepal for de‐identified data. Consent to participate is not applicable as we used de‐identified data of mortality records.

## Data Availability

The project data is kept under the data protection regulation of the HEOC and the Ministry of Health and Population, Nepal and can be obtained with the reasonable request from the first and corresponding author.
